# Insect Visitors of Specialty Cut Flowers in High Tunnels

**DOI:** 10.1093/jee/toac051

**Published:** 2022-04-28

**Authors:** Samantha R Nobes, Judith S Herreid, Karen L Panter, Randa Jabbour

**Affiliations:** Department of Plant Sciences, University of Wyoming, 1000 East University Avenue, Laramie, WY, 82071, USA; Department of Plant Sciences, University of Wyoming, 1000 East University Avenue, Laramie, WY, 82071, USA; Department of Plant Sciences, University of Wyoming, 1000 East University Avenue, Laramie, WY, 82071, USA; Department of Plant Sciences, University of Wyoming, 1000 East University Avenue, Laramie, WY, 82071, USA

**Keywords:** pollinator, natural enemy, high tunnel, cut flower, floral resource

## Abstract

Various strategies incorporate floral resources into agricultural landscapes to support beneficial insects. Specialty cut flower production offers a rarely explored approach to offer floral resources while yielding a marketable product for growers. We characterized insect visitation to six species of specialty cut flowers. Due to Wyoming’s growing conditions, the flowers were grown in high tunnels, thus offering insight into insect abundance in this unique semi-controlled environment. The flower species tested were *Calendula officinalis, Celosia argentea, Daucus carota, Helichrysum bracteatum, Matthiola incana,* and a *Zinnia elegans-Zinnia hybrida* mixture. At least four species were in bloom from early June through late September. The flowers attracted diverse pollinator groups including Diptera, Hymenoptera, Coleoptera, and Lepidoptera. Bees most often visited *Ca. officinalis, H. bracteatum,* and *Celosia spicata* whereas flies most often visited *D. carota*. *Bombus* were the most oft-collected bees from the flowers and were found on all six cut flower species. Wasp abundance varied little across the cut flowers, but wasp community composition was distinct. The highest diversity of wasp families was collected from the *Zinnia* mixture (seven families) in contrast to less diverse collections from *Ce. spicata* (two families). The most abundant wasp families collected were Crabronidae and Sphecidae. Our experiment documented that ornamental cut flower species attract pollinator insects into high tunnel environments. All cut flower species tested were visited by multiple types of beneficial insects. Planting a mixture of specialty cut flowers can support insect diversity while also diversifying on-farm agricultural products through sale of cut flower stems.

Floral resources in managed landscapes support beneficial insects that contribute to pollination, pest control, and overall biodiversity (Goulson et al. 2015, Harris et al. 2016). Effective management strategies that support a diversity of potential pollinators are critical for resilient, productive cropping systems (Rader et al. 2020, Reilly et al. 2020). Including flowers in a foraging area with different shapes, sizes, and colors offers support to greater numbers of pollinators (Mader et al. 2011; Lee-Mäder et al. 2016).

Diverse wildflower plantings on farmland is an oft-researched practice (Albrecht et al. 2021), but grower adoption remains low (Kleijn et al. 2019). Pollinator conservation decisions are poorly understood (Bloom et al. 2021) and potentially driven by economics (Jones Ritten et al. 2017). For example, sales of native wildflower seeds produced in flower strips could provide economic returns exceeding costs of strip establishment and maintenance (Delphia et al. 2019). Recent research expands beyond wildflowers, exploring the effect of ornamental plants (Erickson et al. 2020, 2021) and plant nurseries (Cecala and Wilson Rankin 2021) on bees.

Specialty cut flower production offers another potential strategy to simultaneously enhance pollinator activity and provide economic benefits to growers. The specialty cut flower market—flowers other than roses, carnations, and mums—has grown in the U.S. via cut flowers produced for local markets (Ortiz et al. 2012). Specialty cut flowers cannot stand up to long transportation distances and may have a shorter postharvest life compared to traditional cut flowers (Armitage and Laushman 2008). Production for clients like florists require a variety of floral types for arrangements, inherently offering floral diversity.

Although many cut flowers are produced in open-field settings, they also can be produced in greenhouses and high tunnels (Nobes et al. 2021). Cut flower production in short season, high altitude locations requires protection from frosts, high winds, and large day to night temperature swings. High tunnels are used for season extension and to improve crop yield and quality (Lamont 2009). Modified light and temperature within a high tunnel environment might lead to disorientation or avoidance in pollinators (Leach and Isaacs 2018). Although bumblebees and leafcutter bees can pollinate effectively in covered environments, there may be negative effects on pollinator health that could be mediated by including floral resources in these environments (Kendall et al. 2021). Here, we evaluated pollinator visitation to six ornamental specialty cut flower species, within small-scale high tunnels. We aimed to identify which insects visit cut flowers in high tunnels that could potentially contribute to pollination and pest control in this environment. We hypothesized differences in visitation between flowers given the distinct floral forms.

## Materials and Methods

### Study Site

The experiment was conducted at the University of Wyoming Laramie Research and Extension Center in Laramie, WY (44°45ʹ30″N, 108°46ʹ36″W, 7,200 ft.). The property, approximately 3.5 ha, consists of greenhouses, small research plots, and the student farm. Two high tunnels were used: one oriented east-west and one oriented north-south (Solar Star Greenhouse; Growers Supply, Dyersville, IA). The high tunnels were 12 × 16 ft. with an arch roof style, covered in a double layer of six-mil uninflated polyethylene plastic (Supp. Fig. S1). Tunnels used roll-up sides to provide ventilation and temperature control; sides were opened when temperatures rose above 40°F and closed when temperatures fell below 40°F.

### Cut Flowers

Flowers were grown in spring and summer of 2020 (Nobes 2021). The flowers used were pot marigold *Calendula officinalis* L. ‘Princess Golden’ (Asterales: Asteraceae), stock *Matthiola incana* (L.) W.T. Aiton ‘Lucinda Mix’ (Brassicales: Brassicaceae), strawflower *Helichrysum bracteatum* (Vent.) Andrews ‘Double Mix’ (Asterales: Asteraceae), ornamental carrot *Daucus carota* L. ‘Dara’ (Apiales: Apiaceae), cockscomb *Celosia argentea* ‘Celway Mix’ (Caryophyllales: Amaranthaceae), *Zinnia hybrida* ‘Profusion Yellow’, and *Zinnia elegans* Jacq. ‘Peppermint Stick’ (Asterales: Asteraceae) (Harris Seeds, Rochester, NY). Flowers were selected to span a range of plant families and morphology types used in floral arrangements (see Nobes 2021 for more information on these species).

### Experimental Design

We created plots of each flower species to compare insect visitation between flower species. Within the high tunnels, we set up three blocks each containing one plot of each flower species: six plots × three blocks to yield 18 different plots (Supp. Fig. S2). Each plot consisted of nine potted plants of a single flower species placed in a three by three grid, forming a cluster approximately one meter squared. The *Zinnia* plots contained a mixture of both *Z. elegans* and *Z. hybrida*. Blocks were split between the two high tunnels due to space constraints: the north-south tunnel contained two blocks, one on each side of a central aisle, and the east-west tunnel contained one block with three plots on each side of a central aisle.

### Data Collection

Timed observations recorded the number of unique plant-pollinator interactions during a set amount of time for each flower species. Observations occurred between 10 am and 2 pm approximately every two weeks from 7 June 2020 until 23 September 2020. Observations were made for 15 min in each species plot. A season-long total of 360 min of observation time was spent on each flower species. We used a two-minute transition period between plots to minimize disturbance. During the 15 min observation period, an individual insect was counted a single time even when visiting multiple flowers to determine the number of visiting insects per site. We observed open flowers for insects landing on reproductive flower parts, not stems and leaves. Observations were recorded as bumble bee (Hymenoptera: Apidae *Bombus spp.)*, other native bees (Hymenoptera: Anthophila), honey bee (*Apis mellifera* L. [Hymenoptera: Apidae]), wasps, butterflies and moths (Order Lepidoptera), flies (Order Diptera), and beetles (Order Coleoptera).

Timed collections allowed for a higher level of taxonomic resolution. As with observations, timed collections occurred between 10 am and 2 pm for 15 min per plot on each date. Collections were performed once per month in June, July, and August, and twice in September, for a season-long total of 225 min. Vials or aspirators were used to capture insects found on flowers during the timed period. Collected bees were identified to family and to genus for *Bombus* and *Apis* (Wilson and Messinger Carril 2016). Wasps were identified to family (Goulet and Huber 1993). Flies were identified as Syrphidae or non-Syrphidae (Triplehorn and Johnson 2005).

We recorded bloom counts: the number of open flowers per species at the time of observation according to methods in Nobes 2021. Spent blooms were cut throughout the season to keep the plants in continual bloom.

### Analysis

We tested for the effect of flower species on the season-long total number of insect observations in a particular insect group using general linear models (SAS Institute; Cary, NC), using square-root transformation if needed to meet assumptions. Means separations were conducted with Tukey tests. Bi-partite networks were created with the package *bipartite* (Dormann et al. 2009) in R-project (R Core Team 2018) to visualize the interaction frequency between insects and the cut flower species (e.g., Olsson et al. 2021).

## Results

Overall, we observed flies visiting the specialty cut flowers most often (45%, *n* = 831), followed by bees (25%, *n* = 455), wasps (22%, *n* = 402), beetles (5%, *n* = 88), and butterflies and moths (2%, *n* = 57). *Bombus* spp. were 67% of the total number of observed bees, other native bees comprised 32%, and *A. mellifera* were 1% of the total. Observations of Diptera differed between flower species (*F*_5,12_ = 20.6, *P* < 0.001), with the highest visitation to *D. carota*, more than twice as often as the other flower species (Table 1). Bumble bee visitation differed among flower species (*F*_5,12_ = 32.1, *P* < 0.001), with the highest visitation occurring on *Ca. officinalis*, *H. bracteatum*, and *Celosia spicata*, more often than *M. incana* and *D. carota* (Table 1). For native bees other than bumble bees, observations differed by flower species (*F*_5,12_ = 9.3, *P* < 0.001), with the highest visitation again on *Ca. officinalis*, followed by *H. bracteatum.* Significantly fewer observations of non*-Bombus* native bees occurred on *Ce. spicata, D. carota, M. incana,* and the mixture of *Z. elegans* and *Z. hybrida (*Table 1). The *Zinnia* mixture was visited by wasps more often than *M. incana*; otherwise, wasp observations were similar across the flowers.

**Table 1.** Timed observation abundances (reported as mean ± standard error) accumulated over the season on each flower species

Flower Species	Bumble bees	Other native bees	Wasps	Dipterans
*Calendula officinalis*	29.7 ± 2.9 a	19.0 ± 2.9 a	13.7 ± 2.2 ab	41.7 ± 2.4 bc
*Celosia spicata*	21.0 ± 2.7 ab	4.3 ± 1.2 bc	20.0 ± 2.7 ab	25.0 ± 4.6 c
*Daucus carota*	5.33 ± 0.9 c	4.0 ± 3.0 c	31.7 ± 4.5 ab	102.3 ± 10.1 a
*Helichrysum bracteatum*	26.3 ± 1.8 a	14.7 ± 1.2 ab	27.3 ± 7.7 ab	50.0 ± 4.4 b
*Matthiola incana*	5.7 ± 0.9 c	2.0 ± 1.0 c	8.7 ± 2.9 b	25.7 ± 4.9 c
*Zinnia elegans &* *Z. hybrida*	14.0 ± 0.6 bc	4.3 ± 1.2 bc	32.7 ± 7.3 a	32.3 ± 5.4 bc

Lower-case letters (a, b, c) indicate means comparison according to Tukey of a given insect taxa, compared across the flower species.

**Fig. 1.**
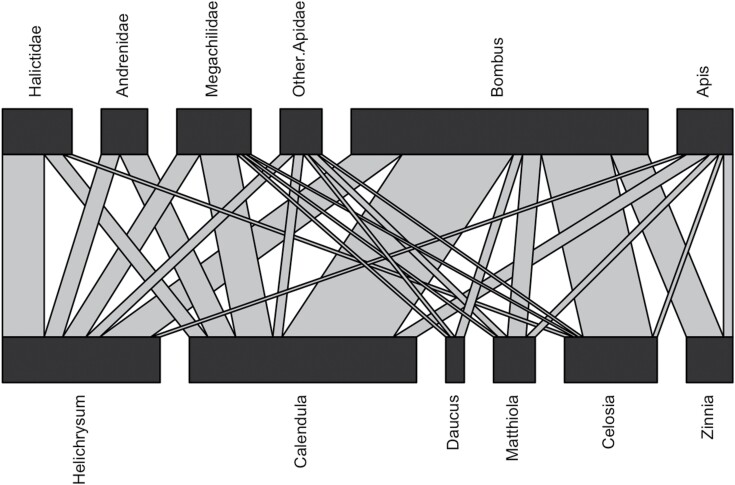
Bi-partite network of bee visitation to the six specialty cut flower species studied. The network was created from 126 bee specimens collected from five sampling periods from mid-June through mid-September. Each flower species was observed for a total of 225 min across these sampling periods.

For Diptera, 42% of collected flies were identified as Syrphidae. The remainder of the flies were nonsyrphids, found on all of the flower species (Supp. Fig. S3). We collected four different bee families: Apidae, Halictidae, Megachilidae, and Andrenidae. *Bombus* were collected from all flower species (Fig. 1). In contrast, adrenids and halictids were only collected from two or three flower species in the experiment, respectively. All bee taxa were collected from *Ca. officinalis* and *H. bracteatum*.

**Fig. 2.**
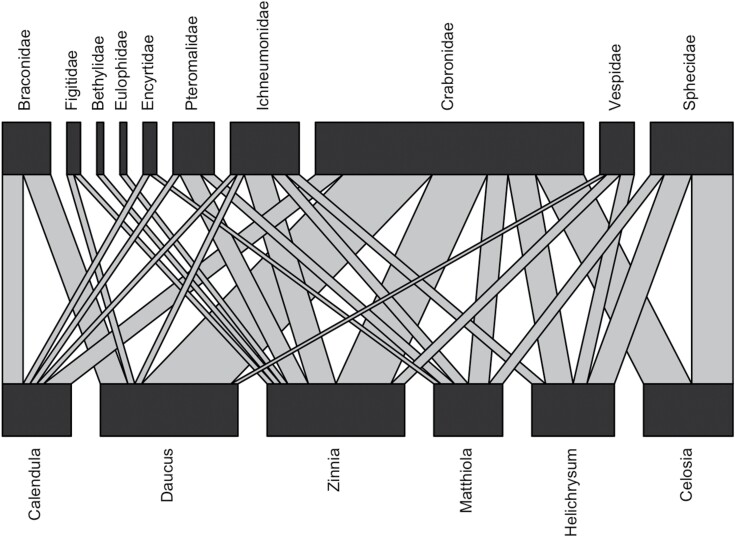
Bi-partite network of wasp visitation to the six specialty cut flower species studied. The network was created from 85 wasp specimens collected from five sampling periods from mid-June through mid-September. Each flower species was observed for a total of 225 min across these sampling periods.

Ten unique wasp families were collected; these families span a diversity of lifestyles ranging from parasitic to predatory (Goulet and Huber 1993, Gibson et al. 1997, Quicke 2014). The most collected wasp families were Crabronidae (45.88%), Sphecidae (14.11%), and Ichnuemonidae (11.76%). Crabronids were collected from all six flower species, whereas sphecids and ichneumonids were collected from five and three flower species, respectively (Fig. 2). Most families (seven) were collected from the *Zinnia* mixture, in comparison to *Ce. spicata*, from which we only collected two families (Fig. 2).

The number of flowers in bloom varied throughout the season with peak number of blooms from mid-August through late September for all flower species (Table 2).

**Table 2.** Total number of flowers in bloom on eight time periods throughout the experiment

	Sample Period							
	1	2	3	4	5	6	7	8
*Ca. officinalis*	7	72	97	81	111	157	334	236
*Ce. spicata*	0	0	61	234	252	262	352	242
*D. carota*	0	0	8	117	280	480	660	225
*H. bracteatum*	2	3	28	51	113	162	339	302
*M. incana*	44	266	497	388	535	732	884	940
*Z. elegans &* *Z. hybrida*	4	80	162	168	313	360	500	190

*Daucus carota*, *Celosia spicata*, and *Matthiola incana* were counted if some of the inflorescence was in bloom. *Calendula officinalis* and *Helichrysum bracteatum* were recorded if the flower head was at least half way opened with the visible centers. 1 (7 June 2020), 2 (22 & 23 June 2020), 3 (6 & 8 July 2020), 4 (21 & 23 July 2020), 5 (4 & 5 August 2020), 6 (15 & 17 August 2020), 7 (3 & 5 September 2020), 8 (22 & 23 September).

## Discussion

Ornamentals used as floral resources can be as attractive or more attractive than native flowers and can provide continual flowering for pollinators (Garbuzov and Ratnieks 2014). In our experiment, specialty cut flowers grown in high tunnels attracted a diversity of beneficial insects. Syrphid and nonsyrphid flies were collected from these flower species (Supp. Fig. S3); both groups can be important to pollination (Orford et al. 2015). Syrphid flies can provide pollination services as adults and pest control services as larvae making them dual service providers (Dunn et al. 2020). Nonbee pollinators may be considered less effective than bees at pollen deposits per flower visit, but they compensate by visiting flowers more often (Rader et al. 2016). In particular, flies, including nonsyrphids, are noted pollinators of importance at high altitudes (Orford et al. 2015, Lefebvre et al. 2018); our experiment occurred at 2195m elevation.

Aculeate wasps, including crabronids, sphecids, and vespids, also have the potential to act as dual service providers through their often generalist predatory behavior and may be ‘backup’ pollinators in certain environments (Brock et al. 2021). Parasitoid wasps provide pest control services in a variety of ecosystems but are generally not thought to be important pollinators (Zemenick et al. 2019).

Two flower species, *H. bracteatum* and *Ca. officinalis*, hosted all bee taxa identified here; species-level identification could reveal more specific relationships. *Ca. officinalis* offers a high reward in both pollen and nectar based on resource per flower (Hicks et al. 2016). The most wasp families were collected from the *Zinnia* mixture. *Z. elegans* also was used in a high tunnel experiment to test methods to improve biological control, and lady beetles and lacewings foraged on *Z. elegans* (Ingwell et al. 2018). *Zinnia* cultivar can affect the attractiveness to different pollinator taxa suggesting that they vary in nutritional reward, floral advertisement, or accessibility (Erickson et al. 2020).

Many species can be grown for cut flower use. Cut flowers must be carefully handled at every stage of production and postharvest, from seeding, planting, harvesting, grading, marketing, selling, and accounting. When we grew these flowers for cut flower stems in past experiments, we harvested multiple times each week (Nobes et al. 2021), in contrast to this experiment where our aim was to support pollinators, and only harvested when flowers were done blooming. We previously observed insect visitation while harvesting, but reduced, particularly in the case of *Matthiola*, which does not regrow after cutting (Nobes 2021). Future research should explicitly examine potential tradeoffs between harvesting and resource provision to bees. Specialty cut flower production can offer an additional crop to market and support a diversity of beneficial insects in a high tunnel setting, and the specific flower choices depend on manager goals (i.e., Anderson et al. 2021).

## Supplementary Material

toac051_suppl_Supplementary_Material
