# Supplementary material for: Insect Visitors of Specialty Cut Flowers in High Tunnels
Source: J Econ Entomol. 2022 Apr 28;115(3):909–13. doi: 10.1093/jee/toac051 (PMC9175293; doi:10.1093/jee/toac051)

Supplementary Figure 1. Two high tunnels 12 by 16 ft. with arch roof style with sides rolled up to provided ventilation.


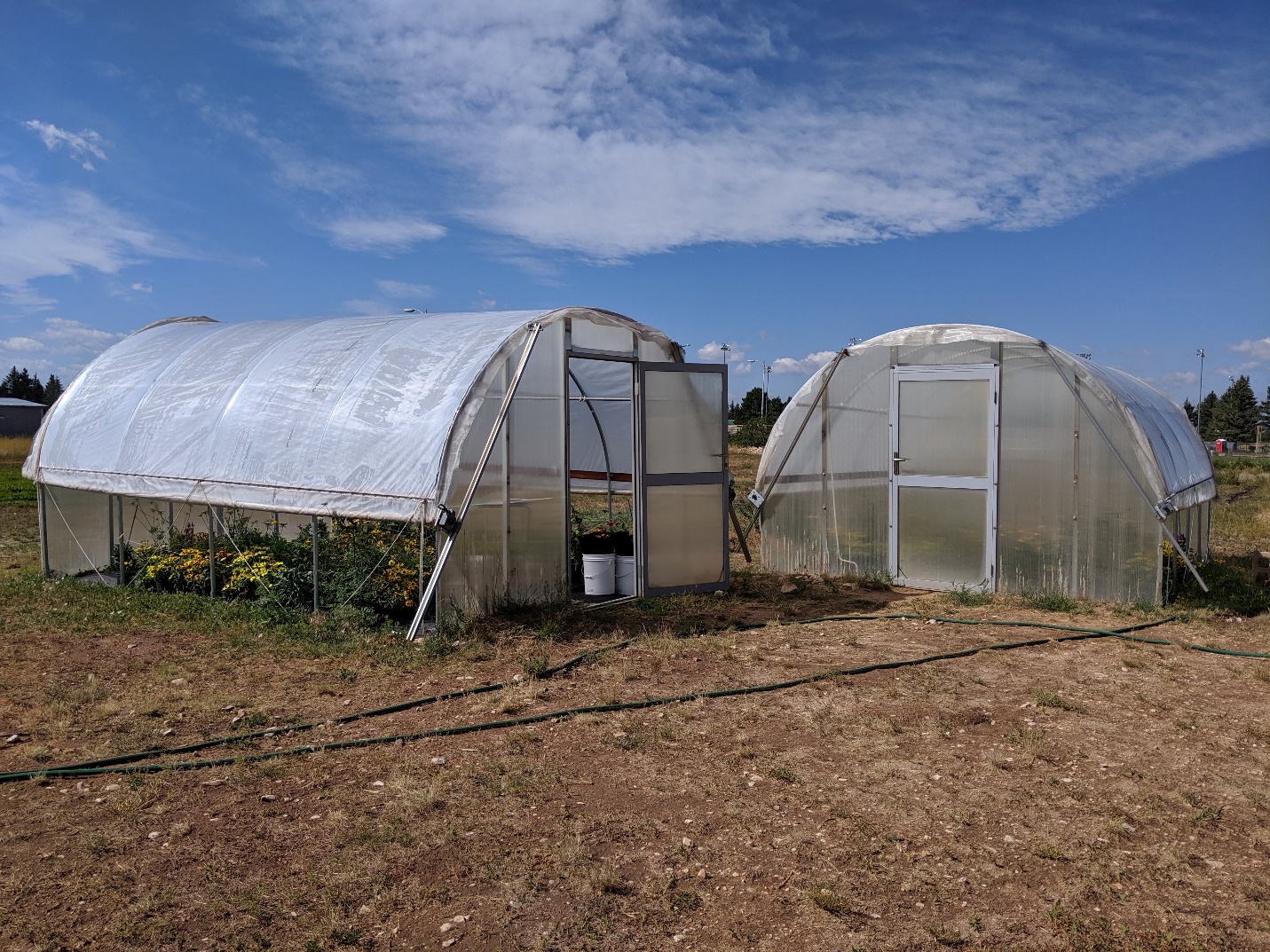


Supplementary Figure 2. Experimental layout in the high tunnels for a total of three replicates per species. This figure if oriented such that North is down.


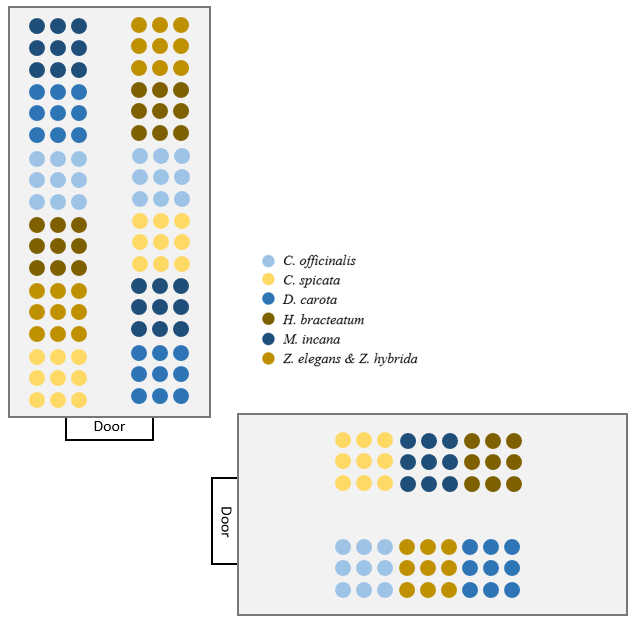


Supplementary Figure 3: Bipartite network of fly visitation to the six specialty cut flower species studies. The network was created from 168 fly specimens collected from five sampling periods from mid-June through mid-September. Each flower species was observed for a total of 225 minutes across these sampling periods.


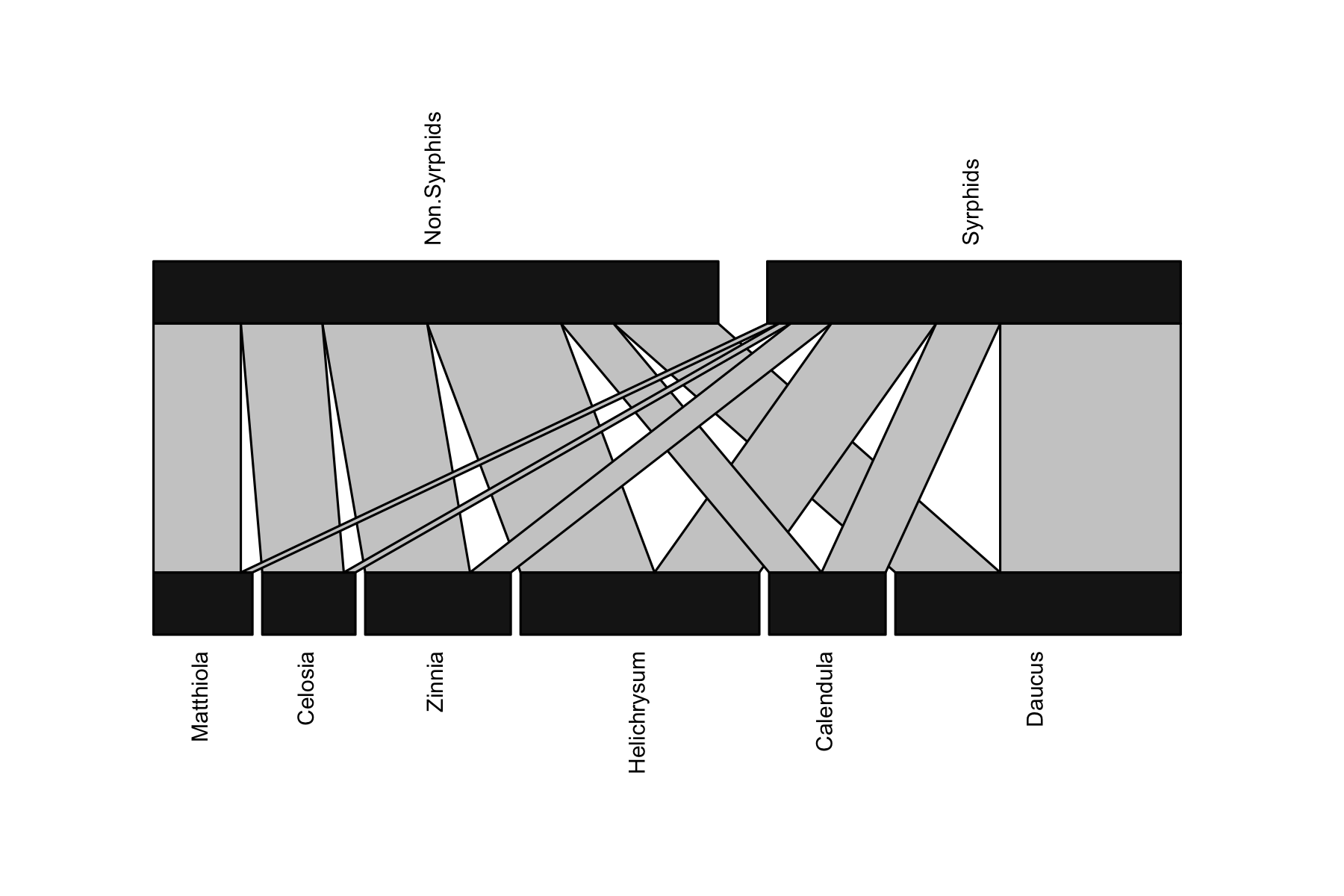

Supplement: toac051_suppl_Supplementary_Material [file toac051_suppl_supplementary_material.docx]
